# Clinically Based Classification and Positioning Indication for Single-Piece Compressive Implants Placement in Regard to Extraction Socket

**DOI:** 10.3390/healthcare10040598

**Published:** 2022-03-22

**Authors:** Mmehul Jani, Vivek Gaur, Anita Gala Doshi, Kiran Patel, Łukasz Pałka

**Affiliations:** 1 Department of Oral and Maxillofacial Surgery, College of Dental Sciences, Ahmedabad 382115, India; janimehul999@gmail.com (M.J.); dr.kp.2557@gmail.com (K.P.); 2 Jaipur Dental College, Maharaj Vinayak Global University, Jaipur 302038, India; drvivekgaur@yahoo.co.in; 3 Dental Health Clinic, Mumbai 400086, India; dranitagala@gmail.com; 4 Private Dental Practice, 68200 Zary, Poland

**Keywords:** one-piece implant, compression screws, root implants, single-piece implants

## Abstract

(1) Background: Dental implantology has been rapidly developing over the last decades. The introduction of new materials, surface modifications and implant designs has brought the need to rethink and systematize our knowledge regarding dental implants. Thus, the aim of this paper is to introduce a new classification and implant positioning indications that can be used to maximize the survival rate and the aesthetic outcome of single-piece compressive screw implants. (2) Materials and methods: This classification was based on a multicenter clinical and radiological observation of 151 patients, in whom 1057 implants were placed with a success rate of 98.5% (1041). The follow-up period was up to 82 months with a mean of 22.34 months. (3) Results: it seems that, in the case of single-piece implants, diameter and length of the implant have influence on their survival rate, whereas smoking and hypertension do not. (4) Conclusions: this paper provides clinicians with comprehensive information about the rationale, criteria and implementation of the new classifications based on a large number of implants and long-term observations.

## 1. Introduction

It is very interesting and, at the same time, may be very confusing that, from the historical perspective, single-piece implants and immediate loading protocol concepts are older than the classical Branemark’s definition of “osseointegration” [1], and we are not talking about a few years or a few decades but hundreds if not thousands of years [2]. The most rapid development in dental implantology has been observed in the last 70 years, together with the introduction of new materials, surface modifications and implant designs [3]. One of the major shifts in this field occurred when clinicians noticed that, at the given primary stability [4], implants can be loaded immediately without the need to wait to achieve secondary stabilization [5]. The next logical step in this development was resigning from multi-element implants (implant, abutment, connection screw) and switching to single-piece implants. This shift from multi-element to single-piece implants resolved some of the potential reasons for marginal bone loss such as bacteria colonization of the gap between the implant and the abutment, which so far had been managed with more or less success with different implant–abutment connection designs or the use of local antiseptics [6,7]. As primary stabilization, associated with high bone mineralization, was the main factor responsible for the success rate in immediate loading implants [8], new surgical procedures were introduced to optimize bone density at the implantation site. These included under-preparation of the implant bed, bone condensation with osteotomes [9] and condensation screws and bicorticalization—anchoring the implant in the second cortical bone [10]. Those techniques of bone utilization have taken root in the minds of practitioners and were generally accepted as effective tools; clinically, they increase the insertion torque and the implant’s primary stabilization. Nevertheless, researchers are still working on reasonable methods of conducting non-destructive 3D bone investigations to visualize and confirm the process of bone modification and its subsequent remodeling.

In the past, single-piece implants were simply recognized as classical implants with abutment permanently attached to the implant’s body, but as our understanding of bone physiology and its reaction to the implant as a foreign body has changed [11,12], a new clinical reality demanded new arrangements. Nowadays, we may risk saying that modern, single-piece compressive implants are the result of combining years of observation and study of bone physiology with more modern biomaterial achievements. They may be used in healed ridges, immediately after tooth extraction or placed in different configurations regarding the extraction socket. They may be used to replace a single tooth or for full-arch rehabilitations with a variety of straight, bendable or angulated abutment shapes and implant prosthetic connections such as a ball, screw-retained, cemented or conometric attachment. All functional and non-functional loading protocols (immediate, early, late) are applicable, depending on the clinical situation.

To properly use these tools and scientific findings regarding single-piece implants and immediate loading protocols for the benefit of our patients, simple and clear clinical guidelines are needed. Thus, the aim of this paper is to introduce a new classification and implant positioning indications that can be used to maximize the survival rate and the aesthetic outcome of single-piece compressive screw implants.

## 2. Materials and Methods

**Figure 1:**
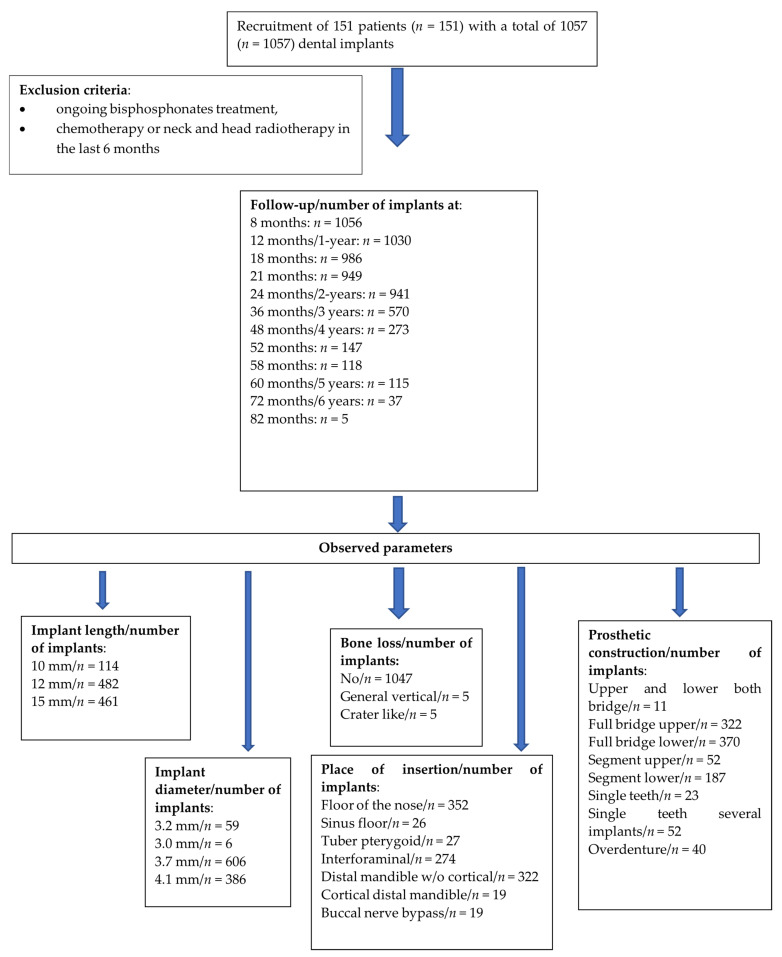
Flowchart of the study design.

This classification was based on a multicenter clinical and radiological observation of 151 patients in whom 1057 implants (KOS, Ihde Dental, Gommiswald, Switzerland) were placed with success rate of 98.5% (1041). In all cases, metal-fused ceramic (MFC) was the material of choice for prosthetic constructions. All patients agreed and signed consent for the study. The follow-up period was up to 82 months with mean 22.34 months as presented in Figure 1 and Table 1. The male-to-female ratio was 80 (53.0%)/71 (47.0%), and the mean patient age was 51.26 + 17.33 (53; 21–91). All data regarding patients’ characteristics are presented in Table 2. During control visits, CBCTs were taken with the use of Vatech Pax-i 3D and transferred to Ez3D-I program for clinical evaluation conducted by experienced surgeons. The exclusion criteria were as follows: ongoing bisphosphonates treatment, chemotherapy or neck and head radiotherapy in the last 6 months. Patients with diagnosed and controlled hypertension, controlled diabetes mellitus, smokers and periodontally compromised were included in the study.

### 2.1. Classification

**Figure 2:**
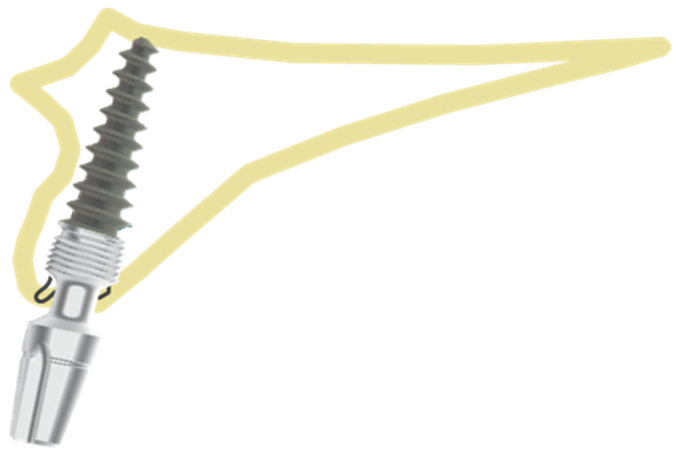
Position 1—implant placed exactly in the socket.

**Figure 3:**
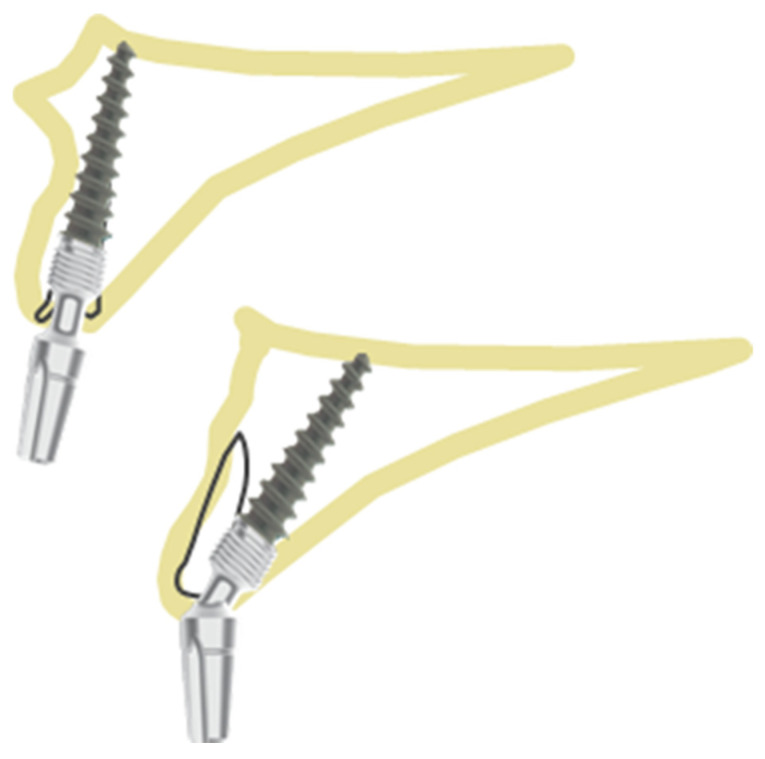
Position 2—an implant placed through palatal/lingual wall of the socket.

**Figure 4:**
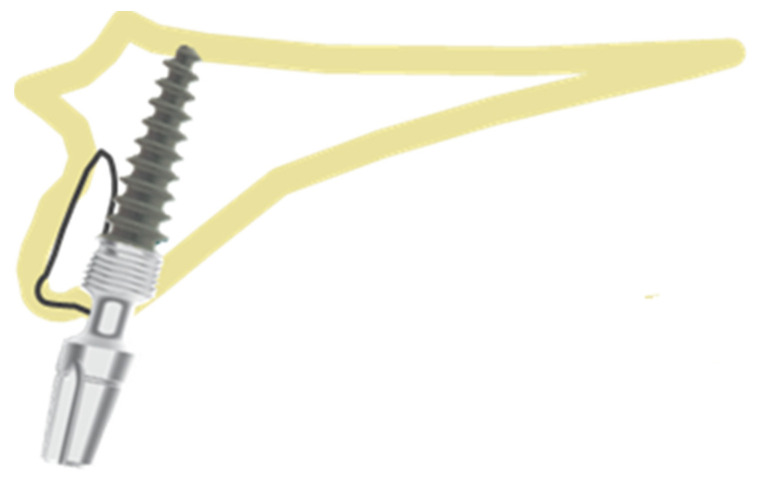
Position 3—an implant placed palatally/lingually in the extraction socket.

**Figure 5:**
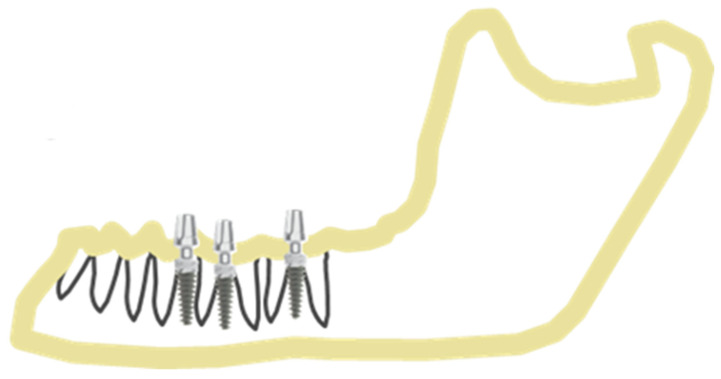
Position 4—an implant placed in the inter-radicular bone.

**Figure 6:**
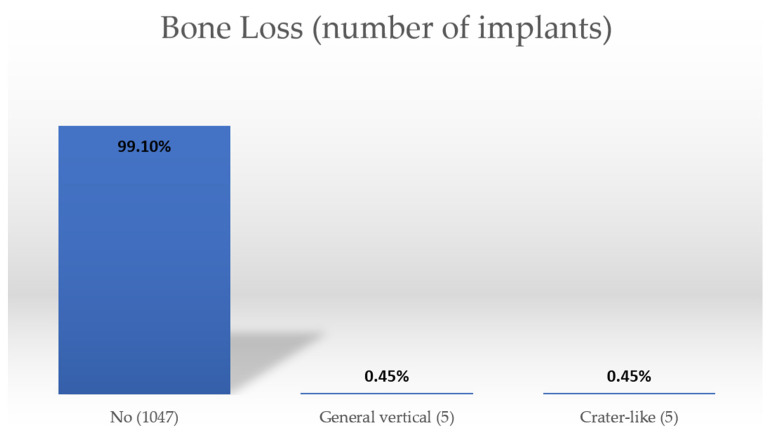
Bone loss.

**Figure 7:**
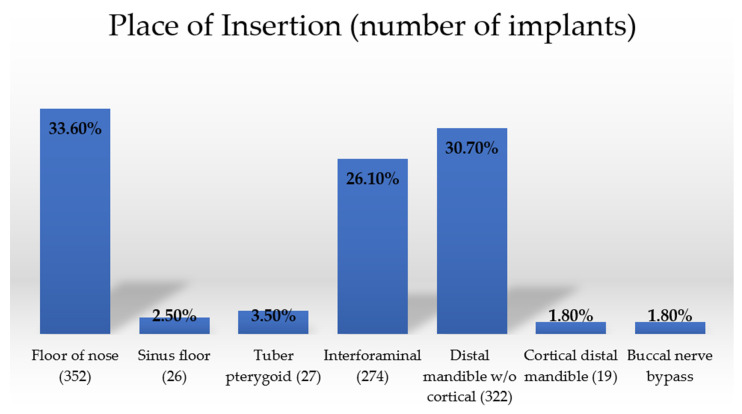
Place of insertion.

**Figure 8:**
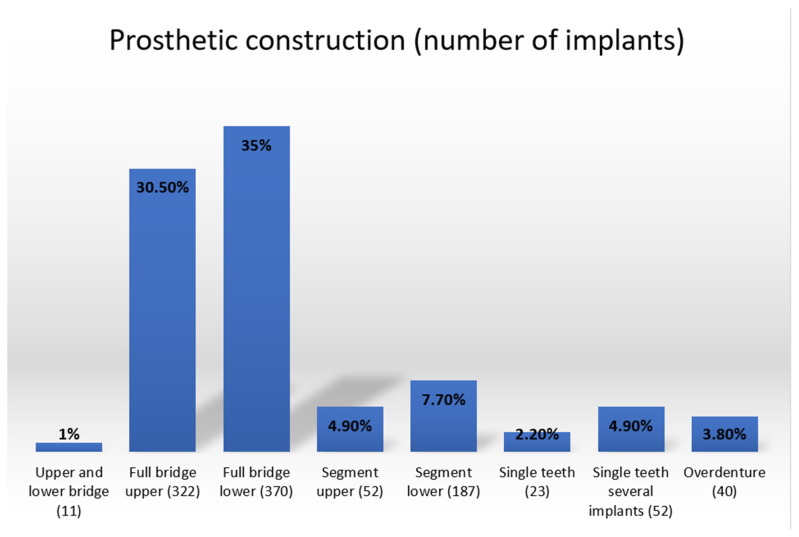
Prosthetic constructions used.

When an implant is placed in the extraction site, it can be anchored in three different bone types that undergo different physiological processes, e.g., remodeling speed and the type and loading thresholds that can influence their resorption. The extraction socket wall consists of lamina cribrosa physiologically designed to connect with periodontal Sharpey’s fibers; thus, this bone starts to resorb immediately after the tooth extraction. The bone surrounding the extraction socket is the cancellous bone consisting of bone trabeculae with spaces filled with marrow or adipose tissue. The most distant bone creating the outer wall from the lingual or palatal side is the cortical bone, which consists of osteons and may be described as highly mineralized with low metabolic activity [13,14]. In the maxillae, there can be additional cortical bone anchorage if the tip of the implant reaches the nasal or maxillary sinus floor. The proposed classification can be divided into two main parts. First part describes the implant positioning in regard to the extraction socket shown in Figure 2, Figure 3, Figure 4 and Figure 5. Second part describes available bone in its vicinity, as shown in Figure 6, Figure 7 and Figure 8.

### 2.2. Implant vs. Extraction Socket Position

Depending on the clinical situation, an implant can be placed directly in the extraction socket, across its palatal or lingual wall or be positioned next to it inter-radicularly with the threads still in contact with its walls. Independently of the conditions and regardless of the presence of additional micro threads, first thread should be placed 1 mm below the bone level.

The entire implant should be surrounded by the socket bone wall with the jumping distance <0.5 mm. Recommended implant diameters are 3.7 mm, 4.1 mm and 5.0 mm (for palatal root of maxillary molars). In cases when even after using a larger implant diameter jumping distance is still >0.5 mm, it is recommended to utilize position number 2 (Figure 3). In cases when, during implant placement, second cortical engagement is possible so that biocritical anchorage is achieved, the operator can bend the implant abutment up to 15–25° if needed.

In this position (Figure 3), the implant is placed through the palatal/lingual wall of the extraction socket. As in the previous one (Figure 2), the entire implant should be surrounded by the bone wall. The jumping distance should be <0.5 mm; that is why the most recommended implant diameter in this case is 3.7 mm. Nevertheless, if it is impossible to properly place the implant so that the rough surface of the implant will not be exposed, position number 3 is advised (Figure 4).

In this position, the implant is placed palatally/lingually to the wall of the extraction socket. The entire implant should be surrounded by the bone wall. The jumping distance should be <0.5 mm. The best diameters are 3.7 mm and 4.1 mm. All threads should be covered with the bone. In this position, bicortical engagement can be easily achieved. Thick palatal/lingual cortical bone will provide better support and infection-resistant environment. It provides good conditions for prosthetic restoration, as more space is provided on the labial side, ensuring nice aesthetic outcome.

In this position (Figure 5), the implant is placed in the interdental or inter-radicular bone and the entire implant should be surrounded by the bone wall.

Recommended diameters are 3.7 mm and 4.1 mm. Notice that all threads should be covered with the bone.

### 2.3. Implant Site vs. Available Bone

Table 3 presents classification of the implant site in regard to the available bone on the buccal and palatal/lingual side. The classification distinguishes 4 different types of clinical situations, depending on the available bone with their survival prediction.

#### 2.3.1. Type I

This type of the clinical situation is when >4 mm bone thickness on the buccal and palatal side is present. It can be characterized as providing excellent survival rate for single-piece implants with very low risk of peri-implant bone loss. The entire implant should be surrounded by the bone and jumping distance should be <0.5 mm. Best positions for the implant placement are 1, 2 and 4. Ideal diameters are 3.7 mm, 4.1 mm and 5.0 mm (the last one is indicated for the palatal socket of the maxillary molars).

#### 2.3.2. Type II

Type II refers to a situation with 2–4 mm bone thickness on the buccal and palatal side. The predicted survival rate in such a case is good. If the implant is placed in the proper position, we can expect very low risk of peri-implant bone loss. Best positions for implant placement are 2 and 3. Ideal implant diameters are 3.2 mm and 3.7 mm.

#### 2.3.3. Type III

Type III refers to a situation where there is 1–2 mm bone thickness on the buccal and palatal side. Survival rate can be described as medium, which means that in such a condition placing implant is risky. However, if the implant is placed in the proper position, the risk of peri-implant bone loss is low. Best position for implant placement in this case is 1. Ideal diameters are 3.0 mm and 3.2 mm. Another possible option in such a situation is to use one-piece bicortical implant with a polished surface.

#### 2.3.4. Type IV

Type IV refers to a situation where the bone thickness is <1 mm on the buccal and palatal side. Survival rate is poor with high chance of bone loss due to peri-implantitis. In such a situation, it is strongly recommended to utilize a one-piece bicortical implant.

## 3. Results

Statistically significant differences in survival rate were observed between males and females and in patients with and without diabetes mellitus, as presented in Table 4, whereas hypertension and smoking do not seem to influence the survival rate.

Statistically significant differences in survival rate were observed between compressive screw implants with different lengths, as shown in Table 5. The survival rates of implants with the length of 10 mm were statistically significantly less successful than compressive screw implants with the length of 15 mm.

The results of the comparison between different diameters of compressive screw implants are shown in Table 6. There are statistically significant differences in survival rate between the observed diameters of implants. Implants with the diameter of 3.2 mm were statistically significantly less successful.

The types and percentage of bone loss around implants are presented in Figure 6. Between different bone losses there are statistically significant differences. Implants without bone loss have statistically significantly better survival rates (Table 7). 

There are no statistically significant differences in implant survival rates in regard to the place of insertion (Figure 7 and Table 8).

There are statistically significant differences in implant survival rate in regard to the prosthetic work restoration range i.e., its length and localization (Figure 8 and Table 9). Segment lower prosthetics as well as single tooth restorations are less successful than other types of implant-retained prosthetics.

## 4. Discussion

Despite quite a low implant failure rate obtained in this study, the evaluation of potential risk factors is of the utmost importance, especially for the practitioners beginning to work with single-piece implants. Since the loading is immediate, there is no time for mistakes. Therefore, the knowledge and experience of other practitioners are so important to ensure long-term success with the best possible functional and aesthetic outcome. Up to date, a very limited number of large studies are available regarding the indications and treatment protocols for single-piece compressive screw implants. In the present study, a new classification has been proposed based on the retrospective data of 1057 implants inserted in 151 patients within a period of 82 months, where such factors as patients’ gender, age, site of implantation, implant length and diameter, type of prosthetic work and patients’ systemic diseases were compared.

In the literature, there are many classifications of bone type, their shape, volume and mineralization in regard to the implant placement and its survival rate [15,16,17,18,19,20]. These classifications, however, are mostly made for two-piece implants with late or classical loading protocols. The unique feature of single-piece compressive implants such as KOS, which have a very thin and bendable implant neck, allows the operator to place them in a manner that would have been impossible with classical implants.

In the present study, the differences between classical, i.e., two-piece, and single-piece implants are visible, especially when it comes to the implant placement. In two-stage implants, the success rate decreased for the posterior mandible and maxillae in comparison with the implants placed in the front [21]. We did not find any statistically significant differences in survival rate between implantation sites. The bone condensation occurring during the implant bed preparation and compressive implant placement allows for achieving very high primary stability, which, together with immediate loading mechanically stimulating the bone, might explain these findings [22,23].

In this study, statistically significant differences in survival rates were observed between males and females, which contradicts the studies on classical two-stage implants described in the literature [24,25].

The factors that seem to influence the survival rate are the type of implant-retained prosthetic work and multiple single-implant restorations. Basically, a segment is considered as a reconstruction consisting of more than one prosthetic tooth unit, and in the ideal situation, it is recommended to place at least three implants for this purpose. This can create a difficult situation, especially in the posterior mandible where the available bone and anatomical structures such as the mandibular nerve canal and mental nerve severely restrict the placement options [21]. So, in order to fulfil the three-implant rule, a long bridge spawn is utilized, which from the mechanical point of view creates higher loading forces and strains [26]. In the presented study, all prosthetic reconstructions were performed with MFC, but the range of possible materials that can be used with this type of implants includes metal acrylic bridges, zirconium, PEEK-based or even graphene-reinforced materials. The possibility to trim or bend the abutment in order to position it in the most desirable place and create enough space for the prosthetic restoration allows for fulfilling all aesthetic and functional requirements. Nevertheless, long-term studies that would compare different prosthetic materials and their impact on implants are needed.

According to the literature, classical implants in tobacco smokers or diabetic patients [27] are considered risk factors for treatment success. In the present study, smoking did not affect the treatment outcome, as there was no statistically significant correlation between implant survival rate and smoking, whereas diabetes myelitis was still a risk factor for the single-piece implant survival rate, which is in accordance with other researchers [28,29].

As the implants used in the study have rough surfaces, when bone loss occurs it will be exposed to the harsh environment of the oral cavity, the bacterial contamination will lead to periimplantitis, and subsequent implant loss can be expected. This can explain the differences in survival rates between implants with and without bone loss, regardless of their type.

In the contemporary literature, the effect of the implants’ geometry on the failure rate is a discussable issue. Some studies showed that small implants failed more often than larger ones [30,31], whereas others could not present any significant correlation between the implant length and survival rate [32]. In the present study, implants of 10 mm in length and 3.2 mm in diameter have been positively related to implant failure, presenting a statistically significant correlation. However, it should be mentioned that this classification is based on the clinical experience of the authors; therefore, future studies are needed to validate this proposal.

## 5. Conclusions

This classification identifies the best possible positions for single-piece implant placement in regard to the extraction socket. Moreover, it may be a useful tool for clinicians during the planning and execution of implant treatment with this type of implants. It offers a novel classification system and clear guidelines that may be utilized to improve the survival rate and aesthetics of prosthetics retained on single-piece compression implants. This way, it fills the very important gap in the field of dental implantology.

## Figures and Tables

**Table 1:** Implant survival rate.

Implant Type	Follow-Up Period (in Month/Year)	No. of Implants with This Follow-Up	Cumulative No. of Failures	Cumulative Survival Rate
Compressive screw	8 months	1056	2	99.8%
	12 months/1 year	1030	2	99.8%
	18 months	986	2	99.8%
	21 months	949	3	99.7%
	24 months/2 years	941	3	99.7%
	36 months/3 years	570	3	99.7%
	48 months/4 years	273	3	99.7%
	52 months	147	4	99.0%
	58 months	118	4	99.0%
	60 months/5 years	115	4	99.0%
	72 months/6 years	37	4	99.0%
	82 months	5	4	99.0%

**Table 2:** Patients’ characteristics.

Observed Parameter		*n* (%)/(X ± SD; (Med; Min-Max))
Number of patients		151
Number of implants		1057
Number of implants in full function		1041 (98.5%)
Age		51.26 ± 17.33 (53; 21–91)
Gender	Male/Female	80 (53.0%)/71 (47.0%)
Hypertension	Yes/No	49 (32.5%)/102 (67.5%)
Diabetes mellitus	Yes/No	63 (41.7%)/89 (58.3%)
Smoker	Yes/No	17 (11.3%)/134 (88.7%)

**Table 3:** Classification of implant site regarding available bone on the buccal and palatal/lingual side.

	Available Bone	Survival Prediction	Best Position	
Type I	>4 mm bone	Excellent	I, II, III	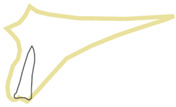
Type II	2–4 mm bone	Good	II, III	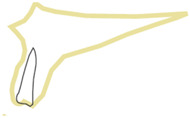
Type III	1–2 mm bone	Medium	I or bicortical implant advised	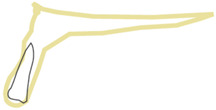
Type IV	<1 mm bone	Low	Bicortical implant advised	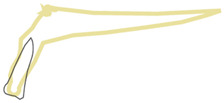

**Table 4:** Implant survival rate and patients’ characteristics.

Observed Parameters		Radiological Follow-Up	Clinical Inspection as Follow-Up	Patient Report as Follow-Up
Overall Survival Rate for all Implants		99.0%	99.0%	99.0%
Gender	Male	100%	100%	100%
	Female	98.0%	98.0%	98.0%
Significance		*p* = 0.027 *	*p* = 0.027 *	*p* = 0.027 *
Hypertension	Yes	100%	100%	100%
	No	98.1%	98.1%	98.1%
Significance		*p* = 0.051	*p* = 0.051	*p* = 0.051
Diabetes mellitus	Yes	100%	100%	100%
	No	97.7%	97.7%	97.7%
Significance		*p* = 0.018 *	*p* = 0.007 *	*p* = 0.007 *
Smoker	Yes/No	100%	100%	100%
	No	98.9%	98.9%	98.9%
Significance		*p* = 0.374	*p* = 0.374	*p* = 0.374

* Statistically significant; Log Rank.

**Table 5:** Implant survival in regard to implant length.

Implant Length (mm)	Frequency (% of All Implants)	Radiological Follow-Up	Clinical Inspection as Follow-Up	Patient Report as Follow-Up
10	114 (10.8%)	98.1%	98.1%	98.1%
12	482 (45.6%)	98.2%	98.2%	98.2%
15	461 (43.6%)	100%	100%	100%
Significance		*p* = 0.006 *	*p* = 0.006 *	*p* = 0.006 *

* Statistically significant; Log Rank.

**Table 6:** Implant survival in regard to implant diameter.

Implant Diameter	Frequency (% of All Implants)	Radiological Follow-Up	Clinical Inspection as Follow-Up	Patient Report as Follow-Up
3.2 mm	59 (5.6%)	96.6%	99.4%	99.4%
3.0 mm	6 (0.6%)	100%	100%	100%
3.7 mm	606 (57.3%)	98.8%	100%	100%
4.1 mm	386 (36.5%)	100%	100.0%	100.0%
Significance		*p* = 0.033 *	*p* = 0.033 *	*p* = 0.033 *

* Statistically significant; Log Rank.

**Table 7:** Type and frequency of bone loss around implants.

Observed Parameters		Radiological Follow-Up	Clinical Inspection as Follow-Up	Patient Report as Follow-Up
Bone Loss	No	99.3%	99.3%	99.3%
	General Vertical	66.7%	66.7%	66.7%
	Crater-like	60.0%	60.0%	60.0%
Significance		*p* < 0.000 *	*p* < 0.000 *	*p* < 0.000 *

* Statistically significant.

**Table 8:** Implant survival rate in regard to insertion place.

Place of Insertion	Radiological Follow-Up	Clinical Inspection as Follow-Up	Patient Report as Follow-Up
Floor of the nose	100%	100%	100%
Sinus floor	100%	100%	100%
Tuber pterygoid	100%	100%	100%
Interforaminal	100%	100%	100%
Distal mandible w/o cortical	97.3%	97.3%	97.3%
Cortical distal mandible	100%	100%	100%
Buccal nerve bypass	100%	100%	100%
Significance	*p* = 0.237	*p* = 0.237	*p* = 0.237

* Statistically significant; Log Rank.

**Table 9:** Implant survival rate in regard to prosthetics reconstruction type.

		Radiological Follow-Up	Clinical Inspection as Follow-Up	Patient Report as Follow-Up
Prosthetic construction type	Full bridge upper	100%	100%	100%
	Full bridge lower	100%	100%	100%
	Segment upper	100%	100%	100%
	Segment lower	95.7%	95.7%	95.7%
	Single tooth	100%	100%	100%
	Single tooth (several implants)	96.2%	96.2%	96.2%
	Overdenture	100%	100%	100%
Significance	*p* = 0.007 *	*p* = 0.007 *	*p* = 0.007 *	

* Statistically significant; Log Rank.

## Data Availability

Not applicable.
